# Quality of life of adolescents with cancer: family risks and resources

**DOI:** 10.1186/1477-7525-8-63

**Published:** 2010-06-28

**Authors:** Lamia P Barakat, Paige L Marmer, Lisa A Schwartz

**Affiliations:** 1 Division of Oncology, The Children's Hospital of Philadelphia, 3400 Civic Center Blvd., Philadelphia, PA 19104, USA; 2 Department of Pediatrics, University of Pennsylvania School of Medicine, 3400 Civic Center Blvd., Philadelphia, PA 19104, USA; 3 Psychology Programs, Philadelphia College of Osteopathic Medicine, 4170 City Avenue, Philadelphia, PA 19131, USA; 4 Division of Oncology, The Children's Hospital of Philadelphia, 3400 Civic Center Blvd., Philadelphia, PA 19104, USA

## Abstract

**Purpose:**

The goal of this study was to evaluate the relative contribution of treatment intensity, family sociodemographic risk, and family resources to health-related quality of life (QOL) of 102 adolescents in treatment for cancer.

**Methods:**

Adolescents and parents completed self-report measures of teen QOL, family functioning, and parent-child bonding. Based on parent report of family sociodemographic variables, an additive risk index was computed. A pediatric oncologist rated treatment intensity.

**Results:**

Simultaneous regression analyses demonstrated the significant contribution of roles in family functioning and quality of parent-child relationship to prediction of psychosocial QOL (parent and teen-reported) as well as parent-reported teen physical QOL over and above the contribution of treatment intensity. Family sociodemographic risk did not contribute to QOL in these regression analyses. In additional analyses, specific diagnosis, types of treatment and individual sociodemographic risk variables were not associated with QOL. Parent and teen ratings of family functioning and quality of life were concordant.

**Conclusions:**

Family functioning, including quality of parent-child relationship, are central and potentially modifiable resistance factors in teen QOL while under treatment for cancer. Even more important than relying on diagnosis or treatment, screening for roles and relationships early in treatment may be an important aspect of determining risk for poor QOL outcomes.

## Background

Research examining the physical, social, and emotional consequences of pediatric cancer and its treatment highlights the significant impact on health-related quality of life (QOL) [1-3]. The preponderance of research on QOL in pediatric cancer focuses on survivors of cancer and young children on treatment. How cancer diagnosis and treatment stressors specifically affect adolescents is not well understood. Adolescence is typically a period of rapid physical, cognitive and psychosocial change that takes place in the context of shifting relationships and roles within the family [4]. Cancer and associated teen and family stressors may challenge adolescent QOL through their impact on normative adolescent developmental tasks (e.g., ability to attend school, engage in activities with peers, participate in extracurricular activities, take on greater responsibilities within their families, in their schools and in their communities) [5,6]. Adolescents with cancer have been identified as being at greater risk than their younger counterparts up to 15 months post-diagnosis, highlighting the importance of examining QOL in adolescents with cancer [5]. Because little is known about family factors that impact the QOL of adolescents with cancer receiving treatment, this study explores the contribution of family sociodemographic risk and family resources, in addition to treatment intensity.

Characteristics of cancer and treatment including type of diagnosis (particularly those that affect the central nervous system; [7,8]), intensity of treatment [3], and phase of treatment [9] have been associated with QOL for children and adolescents. For example, Landolt and colleagues [1] determined that QOL significantly decreases during treatment for childhood cancer, but most aspects of QOL improve as children move off treatment. Wu and colleagues [10] similarly found that adolescents on treatment were more likely to report poorer QOL than those off treatment. Importantly, regardless of phase of treatment, QOL is significantly associated with intensity of treatment and presence of medical complications. Speechley and colleagues [3] found that QOL was associated with type of cancer and treatment received. Cranial radiation, in particular, contributed most to deficits in QOL.

While treatment characteristics are central to QOL, cancer is treated in the context of the family [11] and models linking family risks and resources to health and psychosocial outcomes for youth with chronic conditions have been described. For example, the Family Adjustment and Adaptation Response (FAAR) model highlights how adaptation to chronic childhood illness is explained in the balance of family demands (stressors from individual, family, and community sources) as well as family resources such as positive parent-child relationships, family functioning, and active coping and positive beliefs/attitudes [12]. Integrating family functioning with disease characteristics, intrapersonal variables, and psychosocial factors, the risk-and-resistance model posits an interaction of risks and resources in explaining adaptation to chronic conditions of childhood including QOL [13-15].

Specific risk factors that may be associated with poorer QOL for children and adolescents with cancer include family sociodemographic characteristics related to limited resources and increased stressors (such as parent education, family income, family structure, family size, and ethnic minority status). Risk for adverse health status is greatest among patients with childhood cancer on and off treatment that are female, of low educational level, with low household incomes, and of ethnic minority status [8,16,17]. Moore, Vandivere, and Redd [17] argue for the value of assessing cumulative sociodemographic risk as it can serve as a summary indicator of the multifaceted environments in which children develop.

Conversely, general family functioning may promote positive outcomes for children undergoing stressful circumstances, and family support has been shown to be a vital resource for children and adolescents with cancer [11,18,19]. Orbuch and colleagues [18] examined parent-child relationships and QOL among childhood cancer survivors, focusing on the association between survivors' evaluations of parent-child relations and self-reported QOL. Survivors who reported quality relationships with their mothers and fathers consistently reported a better QOL, especially within the psychological domain. Similarly, Vance and colleagues [20] found that children who self-reported poorer QOL had mothers who endorsed more symptoms of depression and illness-related stressors. Moreover, Eiser, Eiser, and Greco [21] identified that teens with cancer reported better QOL when their parents remained goal-focused instead of protective in their parenting. In many cases, however, parents exhibit greater levels of distress than their children with cancer creating family burden [2,22], and families of children and adolescents with cancer rate themselves as less cohesive and more conflicted than do families of healthy children [23,24]. Thus, the role of positive parent-child relationships and family functioning may be potential sources of resilience, fostering better QOL outcomes for adolescents with cancer [11,25,26].

Despite considerable research describing QOL of patients with childhood cancer both on and off treatment, studies have rarely targeted adolescents or examined the relative contribution of treatment intensity, family sociodemographic risk, and family resources. Therefore, the goal of this study was to evaluate the relative contribution of treatment intensity, family sociodemographic risk, and family resources to health-related quality of life of adolescents on treatment for cancer. Based on the pediatric cancer literature, we hypothesized adolescents with more intense treatments would report poorer QOL. Consistent with risk-and-resistance models, however, family sociodemographic risk factors and family resources (parent and teen reports of roles in family functioning and quality of parent-child relationships) were expected to significantly contribute to QOL. More specifically, sociodemographic risk factors were expected to be associated with poorer QOL, and adaptive family functioning and higher quality of the parent-child relationship were expected to be associated with better QOL among adolescents on treatment for cancer.

## Methods

This paper represents a sub-analysis from a cross-sectional study examining health-related hindrance of goals and psychological well-being of adolescents with cancer. The appropriate Institutional Review Board approved the study protocol.

### Participant Recruitment

Adolescents currently under treatment for cancer and a primary caregiver were recruited from the outpatient clinic and inpatient unit of the cancer center of an East Coast children's hospital. Patients were eligible if they were: (1) 13 to 19 years of age; (2) greater than one month post-diagnosis and currently receiving treatment for a cancer diagnosis; (3) themselves and their caregivers fluent in English; and (4) physically able to complete the measures. Of 133 eligible adolescents approached for the study, 123 agreed to participate and 102 adolescents and a parent completed the forms. Reasons for refusal to participate included parent did not want adolescent to participate (*n *= 4), study was viewed as too much work (*n *= 2), teen had cognitive limitations (*n *= 1), and teen did not feel well (*n *= 1); reason for refusal was not given by 2 potential participants. Participants did not differ significantly from non-participants on age, gender, or ethnic minority status.

### Measures

#### Disease and treatment characteristics

*Intensity of Treatment Rating - 2 (ITR-2) *[27] described treatment based on medical chart review. The ITR-2 is a valid and reliable approach to reflect disease and treatment variables. It provides an objective classification of the cancer diagnosis and treatment experience, categorizing intensity of pediatric cancer treatment from least intensive (Level 1) through most intensive (Level 4). A pediatric medical resident abstracted data from patient records, and a pediatric oncologist classified treatment into one of four groups.

#### Family sociodemographic risk

Caregivers completed questions regarding themselves and their child to collect demographic information such as age, gender, ethnicity, relationship status, education, employment, and income. Because additive models of risk may serve as better indicators of children at risk than considering one risk factor in isolation [17], a *cumulative risk index *was used to summarize presence of family sociodemographic risk in one and two parent families based on patient ethnic minority background, parent education, parent employment status (no parent with full-time employment), number of children living at home (> 3), and family income below the national poverty level based on family size. While scores ranged from 0 to 5, a substantial proportion of the sample (40.6%) had 0 risk factors present. Therefore, this variable was dichotomized into those with no risk and those with risk (1 or more risk factors present).

#### Family Assessment Device (FAD)[28]

Adolescents and their caregivers reported on this 60-item scale of perceived family functioning using the FAD, which is comprised of 7 scales that measure problem solving, communication, roles, affective responsiveness, affective involvement, behavior control, and general functioning. The FAD describes family properties and transaction patterns that distinguish healthy from unhealthy families. Family members rate how well each statement describes their family by selecting from among four alternative responses: strongly agree, agree, disagree and strongly disagree [29], with average score computed. The FAD has high internal consistency across a variety of different types of families [28]. The Roles scale (clarity of role expectations of family members and perceptions of shared responsibilities) was used in analyses as it reflects aspects of adolescent-parent relationships that are negotiated and change as children move through adolescence and take on increased responsibilities [30]; higher scores indicted greater dysfunction. Cronbach's alphas were acceptable for parent (*α *= .78) and marginal for teen (*α *= .65) report.

#### Parental Bonding Inventory (PBI)[31]

The PBI is a self-report 25-item measure with two components of parental care and parental overprotection. The adolescent selects answers on a 4-point Likert-type scale that best apply to their caregiver (and the caregiver selects answers for their teen) from very like, somewhat like, somewhat unlike, and very unlike. The PBI has been shown to be a valid and reliable instrument with long-term stability over time [32]. Internal consistency was high for teen PBI care (*α *= .86), teen PBI overprotection (*α *= .79), parent PBI care (*α *= .78), and parent PBI overprotection (*α *= .73). Higher scores indicate greater endorsement of each component, care or overprotection.

#### Pediatric Quality of Life Inventory (PedsQL)[33]

Both adolescents and their caregivers reported on the child's quality of life using the generic core of the PedsQL, which measures QOL in healthy children and adolescents and those with acute and chronic health conditions. Designed to assess functioning within physical, emotional, social, and school domains, participants use a number rating system which corresponds to 0 if it is never a problem, 1 if it is almost never a problem, 2 if it sometimes a problem, 3 if it is often a problem, and 4 if it is almost always a problem. Items are then reverse scored and scaled to a range of 0 - 100 with higher scores representing better QOL. The measure is valid in that it distinguishes between healthy children and children with acute and chronic health conditions and distinguishes disease severity within a chronic health condition [34]. We used the physical and psychosocial summary scores for this study. Cronbach's alphas for the parent and teen report on the physical and psychosocial subscales ranged from .87 - .91.

### Procedures

Parents and patients were approached either in clinic or during an inpatient admission by the principal investigator (LS) to invite participation. For those interested in participating, parents and adolescents age 18 or 19 provided informed consent/permission, and adolescents younger than 18 provided assent prior to commencement of data collection. Families returned completed packets of measures in person or via the mail.

### Data Analysis Plan

Analyses were conducted using SPSS 17.0. As part of preliminary analyses, variables were described, teen and parent reports of QOL were compared using intraclass correlation coefficients, and correlations of demographic variables (not included in the family sociodemographic risk index), treatment intensity, sociodemographic risk, and family resource variables with QOL were computed to determine covariates (based on *p *< .05) and describe the pattern of correlations among variables of interest. To test the hypothesis, four simultaneous regression equations (with teen and parent report physical QOL and psychosocial QOL as dependent variables) were computed. Correlation and regression analyses were run within reporter (teen, caregiver) to reduce error due to intrafamilial correlation of data [35]. Predictors were ITR, family sociodemographic risk index and the three family variables (FAD roles, PBI care, PBI overprotection). Power was adequate (> .80) based on anticipated small to medium effect sizes, a *p *value of .05, and five variables entered into each regression equation.

## Results

### Participants

The 58 male and 44 female adolescent participants had a mean age of 15.75 years (*SD *= 1.78). Consistent with our clinic populations, most (67.6%) participants identified as Caucasian. The rest were African-American/Black (14.7%), Hispanic (11.8%; 7 White Hispanic; 5 mixed race Hispanic), Asian (2.9%), and more than one unidentified race (2.9%). Regarding cancer diagnosis, time since diagnosis averaged 20.5 months (*SD *= 38.6, range = 1 - 193.4) with 29.4% diagnosed with leukemia, 19.6% with lymphoma, 40.2% with solid tumors, and 10.8% with brain tumors. See Tables 1 and 2 for description of sociodemographic and disease and treatment characteristics of the sample.

**Table 1:** Sample Sociodemographic and Treatment Characteristics (*N *= 102)

*Variable*	*N*	*%*	*Range*
Male Gender	58	57.0%	
Teen Age, *M *(SD)	15.75	1.76	13 - 19
Parent Age, *M *(SD)	45	5.61	28 - 59
Number of Children, *M *(SD)	2.68	1.15	1 - 8
Parent Ethnicity/Race			
Ethnic Minority	33	32.3%	
Family Income (*n *= 94)			
< $40,000	23	24.5%	
$40,000 - $79,999	31	33.0%	
> $80,000	40	42.5%	
Parent Relationship Status			
Married or in Committed Relationship	81	80.2%	
Other--Single, Divorced, Separated, Widowed	21	19.8%	
Parent Level of Education			
Completed 8^th ^Grade or High School Degree	40	39.2%	
Graduated 2 Year College/Technical School	21	20.6%	
Graduated College or Graduate School	41	40.2%	
Parent Work Status Post Cancer Diagnosis (*n *= 101)			
Work Full-time	36	35.6%	
Work Part-time	26	25.7%	
Not employed currently	39	38.6%	
Family Sociodemographic Risk Index--No Risk	41	40.6%	
Relapse	28	27.5%	
ITR			
Moderately Intensive	21	20.6%	
Very Intensive	45	44.1%	
Most Intensive	36	35.3%	

Note: ITR = Intensity of Treatment Rating

**Table 2:** Sample Cancer and Treatment Characteristics (*N *= 102)

*Variable*	*M*	*SD*	*Range*
QOL Teen Report of Physical Health	55.51	26.26	0 - 100
QOL Parent Report of Teen Physical Health	45.28	25.63	0 - 100
QOL Teen Report of Psychosocial Health	64.70	16.20	23 - 100
QOL Parent Report of Teen Psychosocial Health	55.74	17.21	18 - 100
FAD Teen Roles	2.20	0.34	1.36 - 2.82
FAD Parent Roles	2.23	0.44	1.09 - 3.36
PBI Teen Care from Parent	30.00	5.87	7 - 36
PBI Parent Care	31.73	4.22	19 - 36
PBI Teen Overprotection from Parent	13.22	6.52	2 - 29
PBI Parent Overprotection	13.08	5.22	1 - 25

Note: FAD = Family Assessment Device; PBI = Parental Bonding Inventory

### Preliminary Analyses

#### Characteristics of the sample

Based on PedsQL scoring on a scale from 0 to 100 with a mean of 50 [33,34] data on children with cancer suggesting mean scores on physical QOL ranging from 60.5 - 71.2 and psychosocial QOL ranging from 67.1 - 71.3 [36], teen and parent report of teen physical QOL and psychosocial QOL indicated impairment (see Table 2). PBI scores demonstrated high care and average overprotection by teen and parent report compared to published cut-off scores ranging from 24 - 27 for care and 12.5 - 13.5 for overprotection [32]. FAD scores were above the mean score for 'healthy families' of 2.11 and below the mean score for 'unhealthy families' of 2.48, indicating moderate lack of clarity of roles [29].

#### Intraclass correlations

High concordance was found between teen and parent report of FAD roles (*ICC *= .43, *p *= .003), PBI care (*ICC *= .50, *p *< .001), and PBI overprotection (*ICC *= .60, *p *< .001). Teen- and parent-report of teen physical (*ICC *= .65, *p *< .001) and psychosocial (*ICC *= .60, *p *< .001) QOL were also highly concordant.

#### Evaluation of potential covariates

Demographic variables (teen gender, teen age, parent age) were not significantly associated with parent and teen report of teen QOL. Similarly, disease variables (diagnosis [leukemias, lymphoma, solid tumors, brain tumors], time since diagnosis, total treatment [chemotherapy + radiation + surgery + BMT]) were not associated with QOL.

#### Preliminary correlations

Table 3 shows preliminary correlations among variables.

**Table 3:** Correlations of ITR, Family Sociodemographic Risk, and Family Resources with PedsQL Subscales (*N *= 102)

	Teen Report Physical	Teen Report Psychosocial	Parent Report Physical	Parent Report Psychosocial
ITR	-.14 (NS)	.02 (NS)	-.09 (NS)	-.08 (NS)
Family Sociodemographic Risk Index	-.05 (NS)	-.09 (NS)	.14 (NS)	-.16 (.051)*
FAD Parent Roles	---	---	-.19 (.030)**	-.32 (.001)***
FAD Teen Roles	-.09 (NS)	-.27 (.003)***	---	---
PBI Parent Care	---	---	-.24 (.008)***	.07 (NS)
PBI Teen Care	-.10 (NS)	.10 (NS)	---	---
PBI Parent Overprotection	---	---	.09 (NS)	-.05 (NS)
PBI Teen Overprotection	.14 (.083)*	.04 (NS)	---	---

* *p *< .10, ** *p *< .05, *** *p *< .01

Note: ITR = Intensity of Treatment Rating; FAD = Family Assessment Device; PBI = Parental Bonding Inventory

### Regression analyses

#### Teen-report QOL

The full regression model for teen report of physical QOL was not significant *F*(5, 101) = 1.39, *R*^2 ^= .07, *p *= .236 (see Table 4). None of the independent variables made significant contributions to the variance in teen report of physical QOL, but teen FAD roles (*p *= .065) trended to significance as a predictor in the expected direction. That is, clearer family roles and responsibilities (better family functioning) predicted better physical QOL.

**Table 4:** Simultaneous Regression Analyses for Variables Predicting QOL (*N *= 102)

*Teen Report Physical*	*β*	*t*	*p*
**ITR**	-0.12	-1.24	.217
**Family Risk Index**	-0.05	-0.52	.604
**FAD Teen Roles**	-0.21	-1.87	.065*
**PBI Teen Care**	-0.12	-1.01	.316
**PBI Teen Overprotection**	0.15	1.30	.198
			
***Teen Report Psychosocial***	*β*	*t*	*p*
**ITR**	0.02	0.19	.852
**Family Risk Index**	-0.01	-0.12	.902
**FAD Teen Roles**	-0.32	-2.87	.005***
**PBI Teen Care**	0.08	0.62	.536
**PBI Teen Overprotection**	0.20	1.71	.091*
			
***Parent Report Physical***	*β*	*t*	*p*
**ITR**	-0.07	-0.76	.449
**Family Risk Index**	0.10	1.00	.920
**FAD Parent Roles**	-0.33	-3.17	.002***
**PBI Parent Care**	-0.32	-3.08	.003***
**PBI Parent Overprotection**	0.10	0.95	.345
			
***Parent Report Psychosocial***	*β*	*t*	*p*
**ITR**	-0.06	-0.63	.533
**Family Risk Index**	-0.12	-1.24	.219
**FAD Parent Roles**	-0.36	-3.33	.001***
**PBI Parent Care**	-0.05	-0.45	.651
**PBI Parent Overprotection**	0.08	0.71	.483

* *p *< .10, ** *p *< .05, *** *p *< .01

Note: ITR = Intensity of Treatment Rating; FAD = Family Assessment Device; PBI = Parental Bonding Inventory.

The full regression model for teen report of teen psychosocial QOL trended to significance *F*(5, 101) = 2.14, *R*^2 ^= .10, *p *= .068 (see Table 4). ITR, Risk Index, and teen PBI care did not account for a significant portion of the variance in teen report of psychosocial QOL, but teen FAD roles (*p *= .005) was a significant predictor and teen PBI overprotection trended to significance (*p *= .091) with better family functioning and more parental overprotection predicting higher psychosocial QOL.

#### Parent-report QOL

The full regression model for parent report of teen physical QOL was significant *F*(5, 101) = 3.58, *R*^2 ^= .16, *p *= .005 (see Table 4). ITR, Risk Index, and parent PBI overprotection did not account for a significant portion of the variance in parent report of teen physical QOL, but parent FAD roles (*p *= .002) and parent PBI care (*p *= .003) were significant predictors. Better family functioning and more parental care predicted higher physical QOL.

The full regression model for parent report of teen psychosocial QOL was significant *F*(5, 101) = 2.74, *R*^2 ^= .13, *p *= .024 (see Table 4). ITR, Risk Index, parent PBI care and overprotection did not account for a significant portion of the variance in parent report of teen psychosocial QOL, but parent FAD roles (*p *= .001) was a significant predictor in the expected direction of higher family functioning predicting higher psychosocial QOL.

## Discussion

Understanding the impact of cancer and treatment on adolescent QOL is of central importance to the development of family supports and interventions to sustain adolescent development. The most recent, noteworthy research on QOL of child and adolescents being treated for cancer highlights associations among disease and treatment characteristics with QOL outcomes [5]. Yet, these QOL studies seldom attend to family sociodemographic risks and resources or focus on adolescents with cancer. Although we expected treatment intensity and family risks and resources to predict QOL in adolescents with cancer, family resources were the strongest predictors particularly for psychosocial QOL. Results were not consistent and effects were small suggesting that this set of risks and resources is incomplete in explaining QOL. It is important to note, nevertheless, that teen self-report and parent report of teen QOL are more consistent with the family's level of functioning than with the intensity of the treatment for cancer.

Family variables, roles and parent-child relationship quality, were particularly integral to explaining psychosocial QOL (both parent and teen report) but only parent report of teen physical QOL. The reason for this disconnect is a bit perplexing as teen and parent reports of teen physical QOL were consistent as was their report of family functioning. The significant finding that higher care was associated with lower QOL provides a clue to the importance of examining teen-parent relationships in the context of cancer treatment. We did not, however, measure specific components of family management of cancer and engagement in cancer care such as negotiation of medication management, addressing side effects of cancer treatment, and dealing with strains of inpatient treatment. Berg and colleagues [37,38] highlight that multiple aspects of parent-adolescent relationships, including responsibility, monitoring, and support, are associated with disease management. Roles and overprotection were measured generally but our study did not account for other specific aspects of the parent-adolescent relationship that may influence physical QOL.

Despite the frequent utilization of parental proxy reports, research indicates that parent and child report of QOL may not entirely correspond [39,40]. For instance, a study investigating agreement between parental and child report of quality of life indicates a trend in underestimation of mother reports of quality of life of their children [40]. In contrast, for our sample, parents and adolescents tended to report comparable teen QOL outcomes. Yet, reliance solely on parental report may at times produce inaccurate descriptions of adolescents' QOL given variation in the association of family resources with teen and parent-reported QOL. Based on our findings, however, parents may be employed to evaluate QOL for teens too ill to provide their own self-reports.

This study is unique in its focus on adolescents with cancer allowing for focused evaluation of QOL during this challenging developmental period. Moreover, the relatively large and diverse sample of adolescents on treatment provided sufficient power to simultaneously analyze multiple risks (intensity of treatment, family sociodemographic risk) and resources (family functioning, parent-adolescent relationships). There are, however, methodological limitations to consider. Intensity of treatment, based on a reliable and valid measure that uses oncologist ratings of standard treatment protocols for specific diagnoses, did not predict QOL. Importantly, modifications in protocol over time, complications of treatment, and healthcare utilization (inpatient admissions, emergency department visits) may influence QOL but were not addressed. Also, as noted, measures of family functioning and parent-adolescent relationships did not assess specific aspects of how families function around disease management.

## Conclusions

### Implications for Research

Cross-sectional correlations point to the value of considering family resources, yet prospective studies are required to better elucidate the predictive value of family variables. While we expected family sociodemographic risks to contribute to QOL, these risk factors were not associated with family resources or QOL, regardless of whether their associations were examined as individual variables or as a cumulative index. The potential negative impact of lower family education, income, employment, and function may be indirect and dynamic over time. Thus, longitudinal studies that measure specific cancer-related stressors and outcomes overtime may better highlight the potential role of sociodemographic and family risks on QOL outcomes during the cancer trajectory. Moreover, it is essential that the multifaceted nature of parent-adolescent relationships be evaluated as adolescents are negotiating developmental challenges and goals as well as cancer and treatment in the context of the family.

### Implications for Practice

A thorough understanding of cancer treatment experiences [16] can guide future treatment efforts to improve adolescent QOL. Based on findings of the current study, and because disease complications and treatment intensity are rarely modifiable, family function is a potential target of intervention to improve the social ecology of adolescents with cancer and related QOL outcomes. Despite the Children's Oncology Group's expectation to include patient-reported outcomes (i.e. QOL) in all their research protocols [10], few interventions that address QOL have been tested. Barrera and colleagues [5] provide an initial encouraging report on groups for adolescents with cancer designed to address psychosocial and developmental challenges faced by these patients, yet no other published family interventions for adolescents with cancer were identified. With identified, modifiable family correlates of adolescent QOL, the development of family-based interventions to improve adolescent QOL should be prioritized.

## Competing interests

The authors declare that they have no competing interests.

## Authors' contributions

LPB developed the quality of life aims and hypotheses for this manuscript, carried out data management related to family sociodemographic risk index, and oversaw data analysis. She was responsible for manuscript preparation across all sections. PLM conducted literature review for quality of life, participated in data management and analyses, and prepared tables. LAS (PI for parent study) conceived of the parent study, participated in data management and analyses, and reviewed/revised the manuscript. All authors read and approved the final manuscript.
